# Laplacian normalization and bi-random walks on heterogeneous networks for predicting lncRNA-disease associations

**DOI:** 10.1186/s12918-018-0660-0

**Published:** 2018-12-31

**Authors:** Yaping Wen, Guosheng Han, Vo V. Anh

**Affiliations:** 1 School of Mathematics and Computational Science, Xiangtan University, Hunan, 411105 China; 2 Department of Mathematics, Swinburne University of Technology, PO Box 218, Hawthorn, Vic 3122 Australia

**Keywords:** Bi-random walk, Disease similarity network, Laplacian normalization, LncRNA similarity network

## Abstract

**Background:**

Evidences have increasingly indicated that lncRNAs (long non-coding RNAs) are deeply involved in important biological regulation processes leading to various human complex diseases. Experimental investigations of these disease associated lncRNAs are slow with high costs. Computational methods to infer potential associations between lncRNAs and diseases have become an effective prior-pinpointing approach to the experimental verification.

**Results:**

In this study, we develop a novel method for the prediction of lncRNA-disease associations using bi-random walks on a network merging the similarities of lncRNAs and diseases. Particularly, this method applies a Laplacian technique to normalize the lncRNA similarity matrix and the disease similarity matrix before the construction of the lncRNA similarity network and disease similarity network. The two networks are then connected via existing lncRNA-disease associations. After that, bi-random walks are applied on the heterogeneous network to predict the potential associations between the lncRNAs and the diseases. Experimental results demonstrate that the performance of our method is highly comparable to or better than the state-of-the-art methods for predicting lncRNA-disease associations. Our analyses on three cancer data sets (breast cancer, lung cancer, and liver cancer) also indicate the usefulness of our method in practical applications.

**Conclusions:**

Our proposed method, including the construction of the lncRNA similarity network and disease similarity network and the bi-random walks algorithm on the heterogeneous network, could be used for prediction of potential associations between the lncRNAs and the diseases.

## Background

Long non-coding RNAs (lncRNAs) form a new class of important ncRNAs, with length longer than 200nt [1–3]. Accumulating evidences have indicated that a large quantity of lncRNAs play critical roles in many important biological processes such as chromatin modification, transcriptional and post-transcriptional regulation, genomic splicing, differentiation, immune responses, and cell cycle control [1–4]. Mutations and dysregulations of these lncRNAs have been found to be linked to the development and progression of various complex human diseases [2, 3].

Computational models have been developed to predict potential associations between lncRNAs and diseases. Chen et al. [4] had an assumption that functionally similar lncRNAs tend to associate with similar diseases and vice versa. Based on this assumption, Chen et al. [4] proposed a method of Laplacian regularized least squares for lncRNA-disease association (LRLSLDA) to infer human lncRNA-disease associations. LRLSLDA calculates the Gaussian interaction profile kernel similarity for both diseases and lncRNAs based on known lncRNA-disease associations, and computes the lncRNA expression similarity of Spearman correlation coefficient between each lncRNA pair, then utilizes Laplacian regularized least squares in the lncRNA space and disease space to combine the optimal classifiers in these spaces to identify potential associations. LRLALDA is a semi-supervised classification algorithm that does not require negative training samples. However, a major issue of LRLSLDA is how to combine two classifiers and how to select suitable parameters. Chen et al. [5] developed two novel calculation models for lncRNA functional similarity (LNCSIM). Chen et al. [6] proposed a fuzzy measure-based LNCRNA functional similarity computational model(FMLNCSIM). Chen et al. [7] introduced the model of KATZ measure to predict potential lncRNA-disease association.

Based on the fact that non-coding genes are often cooperated in human diseases to predict potential lncRNA-disease association, Peng et al. [8] proposed a new vector to represent diseases, and applied the newly vectorized data for a positive unlabeled learning algorithm to predict and rank disease-related lncRNAs. Ding et al. [9] proposed a model constructing lncRNA-disease-gene tripartite graphs (TPGLDA) includes gene-disease associations and lncRNA-disease associations, and then applied to the process of resource-allocation on tripartite graphs to construct the potential lncRNA-disease association. However, TPGLDA only focuses on unweighted tripartite graphs.

Some models predict novel associations without referring to known associations between lncRNAs and diseases. Chen [10] proposed a model of hypergeometric distribution for lncRNA-disease association (HGLDA) to predict potential lncRNA disease associations. Zhou et al. [11] proposed a rank-based method called RWRHLD, which integrates the miRNA-lncRNA association network, disease-disease similarity network and known lncRNA-disease association network into a heterogeneous network and implementing a random walk with restart on this heterogeneous network to predict novel lncRNA-disease associations. However, RWRHLDA cannot be applied to lncRNAs without a known miRNA interaction partner.

Some computational models have been applied to predict lncRNA-disease associations based on random walk on networks. Chen et al. [12] considered the limitations of traditional random walks with restart (RWR), and proposed a model of improved random walk with restart (IRWRLDA) to predict lncRNA-disease associations. Sun et al. [13] proposed a method of RWRlncD based on global network to predict potential lncRNA disease associations. However, RWRlncD only considers lncRNAs which have known associations with the disease and ignores lncRNAs that are currently not associated with the disease. Considering the differences in the network topology of lncRNA and disease, Gu et al. [14] proposed a random walk model on global networks for predicting lncRNA-disease associations (GrwLDA). Yu et al. [15] proposed a model that performs bi-random walks to predict lncRNA-disease associations (BRWLDA). However, BRWLDA only considers the semantic similarity of the disease, and the transitional probability between diseases is only empirically estimated.

In this study, we propose a novel computational model of Laplacian normalization and bi-random walks on heterogeneous networks for predicting lncRNA-disease associations (Lap-BiRWRHLDA). Firstly, the method calculates the Gaussian interaction profile kernel similarity of lncRNAs and diseases by known lncRNA-disease associations. Next, we integrate the two sources of similarity to construct an lncRNA-lncRNA similarity network. The disease-disease similarity network can be constructed by the profile kernel similarity of diseases. Subsequently we perform Laplacian normalization on the similarity matrices of lncRNAs and diseases as the transpose matrices. Furthermore, we apply random walks on the lncRNA similarity network and the disease similarity network, respectively. Finally, we use a weighted average of random walks on both networks as a predictor of lncRNA disease associations. We believe that the higher scores of lncRNA-disease associations will have greater possibility for further verification. To evaluate our proposed method, we utilize leave-one-out cross-validation experiments to demonstrate its superior performance compared with existing approaches. Furthermore, the analyses of three cancers (namely, breast cancer, lung cancer, and liver cancer) effectively support the practical application of our method. We then use Lap-BiRWRHLDA to infer potential lncRNA-disease associations. Some high-score results are successfully verified by the LncRNADisease and Lnc2Cancer databases.

## Results

### Leave-one-out cross-validation

To assess the performance of our proposed method, we use the leave-one-out cross-validation to perform the assessment. We leave out each known lncRNA-disease association in turn as test sample, while other known relationships are used as training samples and all unknown relationships are taken as candidate samples. Since disease similarity and lncRNA similarity depend on the Gaussian interaction profile kernel similarity of the known lncRNA-disease association, the disease similarity and lncRNA similarity will change when we delete a known lncRNA-disease association, so we will get different similarities.

A receiver-operating characteristics (ROC) curve is applied to determine the predictive performance, which plots the correlation between true-positive rate (TPR) indicating sensitivity and false-positive rate (FPR) indicating specificity at different thresholds. Sensitivity represents the percentage of the left-out associations achieving the ranking higher than a given threshold; specificity means the percentage of candidate associations achieving the ranking lower than this given threshold. When we vary thresholds, we will obtain the corresponding different TPRs and FPRs. In this way, ROC is drawn and AUC is calculated. As a result, Lap-BiRWRHLDA achieved the AUC of 0.8409, 0.8527 and 0.8429 for three datasets used, respectively.

### The effect of parameters in Lap-BiRWRHLDA

Parameter *α* controls the probability of the random walk restart. To optimize the parameter *α*, we increased *α* from 0.1 to 1 with step size 0.1, and then calculated the corresponding AUC value by LOOCV. After experimental verification, we chose *α*=0.9, and we achieved the AUC values of 0.8409. The experimental results indicate that Lap-BiRWRHLDA offers better performance on the LncRNADisease dataset on October 2012, when *α*=0.9 is selected. Similarly we achieved the AUC values of 0.8527 (*α*=0.2) and 0.8429 (*α*=0.8) based on Lnc2Cancer dataset on July 2016, and the LncRNADisease dataset on April 2016.

### Performance comparison with other methods

**Fig. 1:**
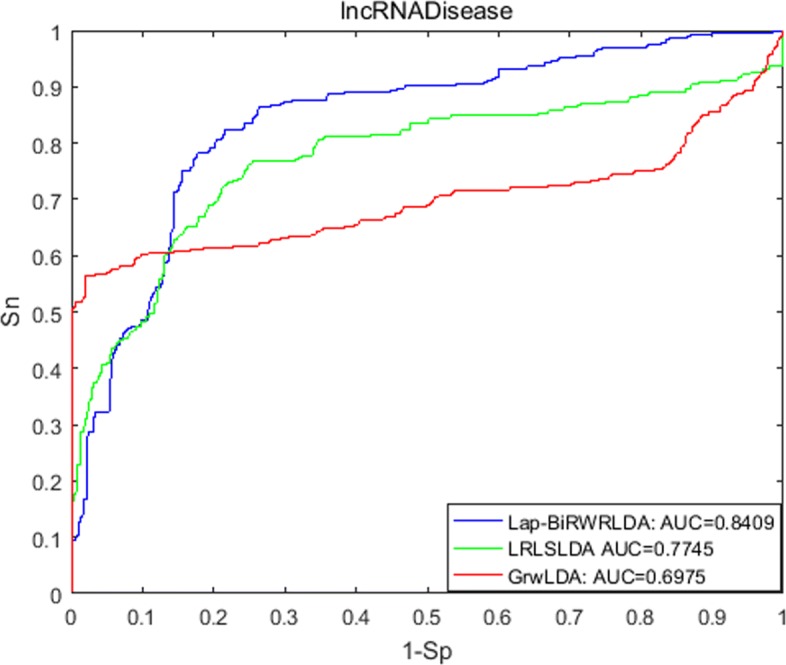
Performance comparison between La-BiRWRHLDA, LRLSLDA and GrwLDA based on the LncRNADisease dataset on October 2012

**Fig. 2:**
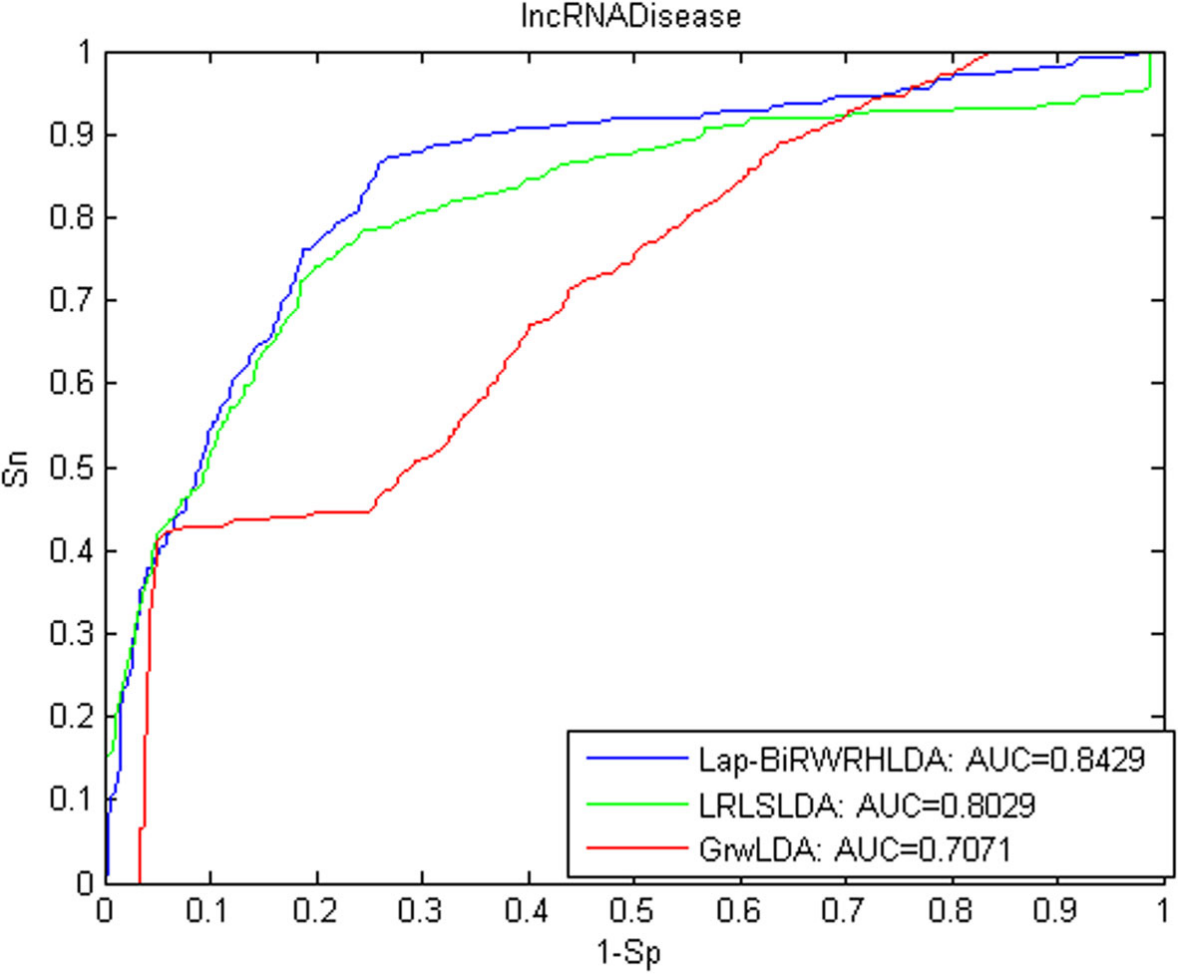
Performance comparison between La-BiRWRHLDA, LRLSLDA and GrwLDA based on the LncRNADisease dataset on April 2016

**Fig. 3:**
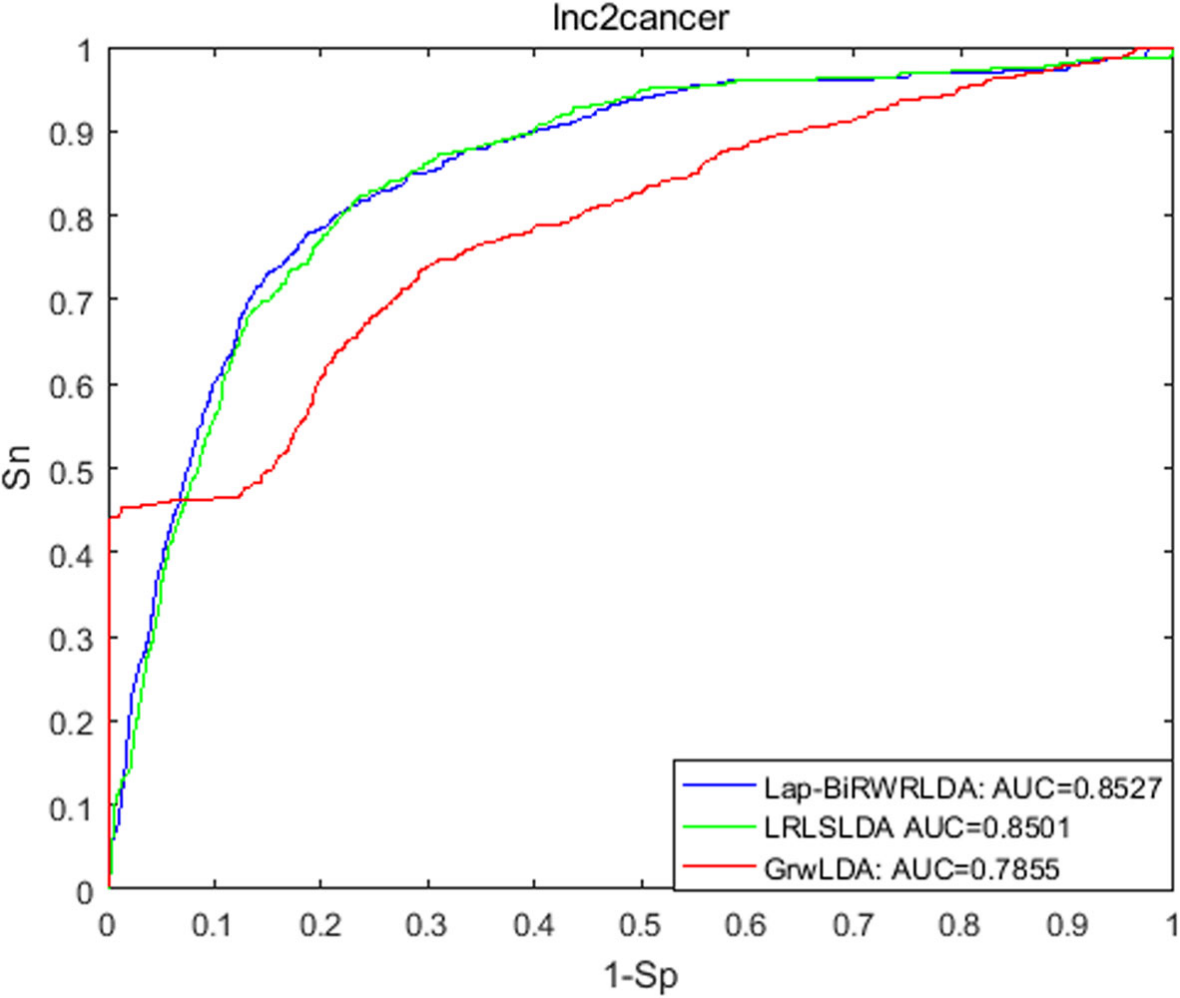
Performance comparison between La-BiRWRHLDA, LRLSLDA and GrwLDA based on the Lnc2Cancer dataset on July 2016

We compared Lap-BiRWRHLDA with previous published methods in LOOCV based on the LncRNADisease dataset on October 2012. (1) LRLSLDA [4] computes Gaussian interaction profile kernel similarity for both diseases and lncRNAs from known lncRNA-disease associations and lncRNA expression profiles, and then applies the framework of Laplacian regularized least squares to identify potential associations. (2) GrwLDA [14] predicts potential associations by a random walk model on global networks for predicting lncRNA-disease associations (GrwLDA). The comparison is shown in Fig. 1. We also compared our method, LRLSLDA and GrwLDA in LOOCV based on the LncRNADisease dataset on April 2016. The comparison is shown in Fig. 2. Figure 3 shows the comparison of Lap-BiRWRHLDA, LRLSLDA and GrwLDA in LOOCV based on the Lnc2Cancer dataset on July 2016. These comparisons consistently indicate a better performance of our method over the state-of-the-art methods for predicting lncRNA-disease associations.

We also compared the LncRNADisease dataset on April 2018 with the LncRNADisease dataset on October 2012, then selected 50 lncRNA-disease associations which were unverified in the LncRNADisease dataset on October 2012 but were verified in the LncRNADisease dataset on April 2018. We compared our method with LRLSLDA, GrwLDA by independently testing the ranking of the 50 relationships. Through experimental tests, our method has 30 rankings higher than the LRLSLDA method, and 41 rankings higher than the GrwLDA method.

### Case studies

To further highlight the performance of Lap-BiRWRHLDA, we studied the predictive performance of three cancers: breast cancer, lung cancer, and liver cancer. For each type of cancer, we take the top 10 most probable lncRNAs as candidates associated with this cancer. Next, we manually checked these lncRNAs by mining biomedical literature from the LncRNADisease dataset and the Lnc2Cancer dataset.

Breast cancer is the second leading cause of female cancer deaths, comprising 22% of all cancers in women [16, 17]. Lap-BiRWRHLDA identifies potential lncRNAs associated with breast cancer and six of the top 10 verified by the recent LncRNADisease dataset. The list in Table 1 shows the lncRNAs associated with breast cancer. Lung cancer is one of the fastest increases in morbidity and mortality, and one of the greatest threats to human health and life. In the past 50 years, many countries have reported that the incidence and mortality of lung cancer are significantly higher. The incidence and mortality of male lung cancer account for the first place among all malignant tumors. Lap-BiRWRHLDA identifies eight out of the top 10 verified (see Table 2). Liver cancer is the fifth most commonly diagnosed cancer and the second most frequent cause of cancer deaths in men worldwide [18, 19]. Lap-BiRWHLDA correctly identifies five liver cancer related lncRNAs. Table 3 lists the lncRNAs related to liver cancer. From these case studies, we can conclude that Lap-BiRWRHLDA is a powerful tool for predicting lncRNA-disease associations with a high level of reliability.

**Table 1:** Breast cancer associated lncRNAs in the top 10 ranking list of Lap-BiRWLDA

Cancer type	lncRNA	Rank	Evidence
Breast	MALAT1	1	LncRNADisease,Lnc2Cancer
Breast	BCYRN1	2	LncRNADisease
Breast	H19	3	LncRNADisease,Lnc2Cancer
Breast	PVT1	5	LncRNADisease,Lnc2Cancer
Breast	NEAT1	6	Lnc2Cancer
Breast	TUG1	8	Lnc2Cancer

**Table 2:** Lung cancer associated lncRNAs in the top 10 ranking list of Lap-BiRWLDA

Cancer type	lncRNA	Rank	Evidence
Lung	PVT1	1	Lnc2Cancer
Lung	H19	2	LncRNADisease,Lnc2Cancer
Lung	MALAT1	3	LncRNADisease,Lnc2Cancer
Lung	HOTAIR	4	LncRNADisease,Lnc2Cancer
Lung	BCYRN1	5	LncRNADisease
Lung	UCA1	6	lnc2Cancer
Lung	GAS5	7	lnc2Cancer
Lung	MEG3	9	LncRNADisease,Lnc2Cancer

**Table 3:** Liver cancer associated lncRNAs in the top 10 ranking list of Lap-BiRWLDA

Cancer type	lncRNA	Rank	Evidence
Liver	H19	1	LncRNADisease,Lnc2Cancer
Liver	MALAT1	2	LncRNADisease,Lnc2Cancer
Liver	HOTAIR	5	LncRNADisease
Liver	UCA1	7	Lnc2Cancer
Liver	MEG3	9	Lnc2Cancer

## Discussion

Accumulated experimental evidences have shown that lncRNAs play an important role in the human complex disease mechanism, and mutations or disorders of lncRNAs are associated with various complex diseases. More and more evidences show that it is crucial to propose an effective computational model to infer potential lncRNA-disease associations. In this article, we proposed a novel computational model of Laplacian normalization and bi-random walks on heterogeneous networks for predicting lncRNA-disease associations. Our method shows better performance in LOOCV experiments by comparison with previous methods. In 50 unverified lncRNA-disease associations experiments, We compared our method with LRLSLDA, GrwLDA. The results indicated that our method has the higher ranking. Furthermore, the study of the cases of breast cancer, lung cancer, and liver cancer shows that our method improves the performance of predicting potential relationships.

Although our method can improve the prediction accuracy, it still has some limitations. For example, construction of the disease-disease similarity matrix relies on the Gaussian interaction profile kernel similarity matrix for diseases from the known disease-lncRNA associations. In further work, we will improve our method in the following aspects: Firstly, Lap-BiRWRHLDA relies on the calculation of similarity matrix when constructing an lncRNA similarity network, and so the incompleteness of data may affect the final performance. Therefore, the integration of gene disease correlation data or the addition of more bioinformatics data may improve the performance of our method. These aspects have been considered in previous methods such as TPGLDA [9] and BRWLDA [15]. Secondly, the bi-random algorithm performs random walk restarts on lncRNA similarity networks and disease similarity networks separately; how to better integrate random walks on two networks is an issue in our future research.

## Conclusions

In this study, we proposed a method called Lap-BiRWRHLDA to predict the relationship between lncRNA and diseases. This model utilizes the Laplacian normalization of the lncRNA similarity matrix and the disease similarity matrix. Then constructs a heterogeneous network based on lncRNA similarity network, disease similarity network and available lncRNA-disease associations. Next, it applies bi-random walks on the heterogeneous network to predict potential associations between lncRNAs and diseases. Our method can be used to better identify potential associations between lncRNAs and diseases.

The reason why our method has good results is mainly due to two factors. On the one hand, we exploit the similarity of lncRNAs by integrating Gaussian interaction profile kernel similarity of lncRNA and lncRNA expression similarity, and then apply Laplacian normalization. We also rely on lncRNA similarity matrices to construct an lncRNA similarity network. On the other hand, the bi-random walk algorithm simulates random walk restarts on the lncRNA similarity network and disease similarity network; we then infer the relationship between lncRNAs and diseases by weighted averaging. We believe that the higher the score of potential lncRNA-disease relationship is, the higher the probability of association is.

## Methods

### Data sets

We downloaded three data sets of lncRNA-disease associations from the supplementary files of published articles [4, 8], which contains 293 experimentally confirmed lncRNA-disease relationships between 167 diseases and 118 lncRNAs from the LncRNADisease database on October 2012 [4], 454 known lncRNA-disease associations between 162 diseases and 187 lncRNAs from the LncRNADisease database on April 2016, and 594 lncRNA-disease associations between 79 diseases and 310 lncRNAs from the Lnc2Cancer database on July 2016 [8]. The adjacency matrix of lncRNA-disease associations is denoted as *A*, where the value *A*(*i*,*j*) of row *i* and column *j* is 1 if disease *d*(*i*) is related to lncRNA *l*(*j*), otherwise it is 0. Let *L*={*l*(1),*l*(2),⋯,*l*(*nl*)} denote the set of lncRNAs, and *D*={*d*(1),*d*(2),⋯,*d*(*nd*)} denote the set of diseases.

We also downloaded lncRNA expressions and the gene expression levels from the supplementary files of the published articles [4, 8], which contain 21626 expression profiles across 22 human tissues or cell types and 60245 gene expression levels in 16 tissues. Let set *L*_1_, where *L*_1_ is composed of lncRNAs with lncRNA expression profiles (*L*_1_⊆*L*). According to the previous approaches [4], if *l*(*i*), *l*(*j*)∈*L*_1_, we calculated the Spearman correlation coefficient of *l*(*i*) and *l*(*j*) as the lncRNA expression similarity. The lncRNA expression similarity matrix is represented by matrix *SPC*, where *SPC*(*l*(*i*),*l*(*j*)) is the expression similarity between *l*(*i*) and *l*(*j*) if they belongs to *L*_1_, otherwise 0.

### Laplacian normalization

Suppose that *M*=*M*(*i*,*j*),*i*,*j*=1,2,⋯,*N*, is a symmetric matrix, *D* is a diagonal matrix of which *D*(*i*,*i*) is the sum of row *i* of *M* and *D*(*i*,*j*)=0 for *i*≠*j*. *M* is normalized by $\hat {M} =D^{-1/2}MD^{-1/2}$, which also yields a symmetric matrix. The elements of $ \hat {M}$ are defined by

1 $$ \hat{M}(i,j)=\frac{M(i,j)}{\sqrt{D(i,i)D(j,j)}}  $$

This process is called Laplacian normalization of *M*. It is often used to normalize a weighted matrix of a network [4, 20, 21].

### Construction of the lncRNA-lncRNA similarity matrix

Based on the assumption that similar diseases tend to show a similar interaction or non-interaction with the lncRNAs, the Gaussian interaction profile kernel similarity of lncRNAs can be calculated from known lncRNA-disease associations [4]. The lncRNA interaction profile *IP*(*l*(*i*)) is a binary vector which is 1 if lncRNA *l*(*i*) is related to the disease, 0 otherwise, defined as the *i*-th column of the adjacency matrix *A* of the known lncRNA-disease association network constructed above. Then we can calculate the Gaussian interaction profile kernel similarity of lncRNA *l*(*i*) and lncRNA *l*(*j*) from their interaction profiles as

2 $$ KL(l(i),l(j))=exp\left(-\gamma_{l}\Vert IP(l(i))-IP(l(j))\Vert^{2}\right),  $$

where the parameter *γ*_*l*_ controls the kernel bandwith, which is calculated based on the new kernel bandwidth parameter $\gamma _{l}^{\prime } $ as follows:

3 $$ \gamma_{l}=\gamma_{l}^{\prime }/\left(\frac{1}{nl}\sum_{i=1}^{nl}\Vert IP(l(i))\Vert^{2}\right),  $$

where *nl* denotes the number of lncRNAs. For simplicity we set $\gamma _{l}^{\prime }=1$ as in the previous works [4, 22].

Following previous approaches [4], we construct the similarity of lncRNAs by combining the lncRNA expression similarity and Gaussian interaction profile kernel similarity. We denote by *SL* the lncRNA similarity matrix, where the element *SL*(*i*,*j*) defines the similarity between lncRNA *l*(*i*) and lncRNA *l*(*j*) as

4 $$ {\begin{aligned} SL(l(i),l(j))=\left\{ \begin{array}{cc} ew\cdot {SPC(l(i),l(j))}+(1-ew)\cdot {KL(l(i),l(j))}, &\text{if \({l(i)},{l(j)}\in L_{1}\)} \\ KL(l(i),l(j)), & \text{otherwise} \end{array} \right. \end{aligned}}  $$

where *SPC*(*l*(*i*),*l*(*j*)) represents the expression profile similarity of lncRNA *l*(*i*) and lncRNA *l*(*j*), and its value is the Spearman correlation coefficient of lncRNA *l*(*i*) and lncRNA *l*(*j*), so the matrix *SPC* is a symmetric matrix. *KL*(*l*(*i*),*l*(*j*)) represents the Gaussian interaction profile kernel similarity of lncRNA *l*(*i*) and lncRNA *l*(*j*), so the matrix *KL* is also a symmetric matrix. Therefore the lncRNA similarity matrix *SL* is a symmetric matrix. In Eq. (4), *ew* is the weight coefficient of lncRNA expression similarity; for simplicity we set *ew*=1/2.

Next, using Laplacian normalization, the element *SL*(*i*,*j*) is calculated through two steps:

5 $$ LL^{\prime }(i,j)=\left\{ \begin{array}{cc} \frac{SL(i,j)}{\sqrt{\sum {_{i}SL(i,j)}\sum {_{j}SL(i,j)}}}, &\text{\(SL(i,j)\neq{0}\)} \\ 0, & \text{otherwise} \end{array} \right.  $$

6 $$ LL(i,j)=\left\{ \begin{array}{cc} \frac{LL^{\prime }(i,j)}{\sum {_{j}LL^{\prime }(i,j)}}, &\text{\(SL(i,j)\neq{0}\)} \\ 0. & \text{otherwise} \end{array} \right.  $$

### Construction of the disease-disease similarity matrix

Similar to lncRNAs, the Gaussian interaction profile kernel similarity of diseases can be constructed as

7 $$ KD(d(i),d(j))=exp\left(-\gamma_{d}\Vert IP(d(i))-IP(d(j))\Vert^{2}\right).  $$

Here *IP*(*d*(*i*)) is defined as the *i*-th row of the adjacency matrix *A* of the known lncRNA-disease association. It is a binary vector representing the relationship between disease *d*(*i*) and each gene. The Gaussian interaction profile kernel similarity matrix *KD* is a symmetric matrix. The parameter *γ*_*d*_ is calculated as

8 $$ \gamma_{d}=\gamma_{d}^{\prime }/\left(\frac{1}{nd}\sum_{i=1}^{nd}\Vert IP(d(i))\Vert^{2}\right),  $$

where *nd* denotes the number of diseases; for simplicity we set $\gamma _{d}^{\prime }=1$ as in the previous works [4, 22].

From relevant research [4, 19], to improve the predictive accuracy of disease similarity, we apply the logistic function transformation to represent the similarity of diseases. The disease similarity is redefined as

9 $$ SD(d(i),d(j))=\frac{1}{1+\exp ({c\cdot {KD(d(i),d(j))+d}})},  $$

where *c* and *d* are two parameters, for which we adopt the same parameter selection as in the previous studies [4, 19], i.e. *c*=−15,*d*= log(9999). The disease similarity matrix *SD* is a symmetric matrix. Next, using Laplacian normalization, the element *SD*(*i*,*j*) is calculated through two steps:

10 $$ LD^{\prime }(i,j)=\left\{ \begin{array}{cc} \frac{SD(i,j)}{\sqrt{\sum {_{i}SD(i,j)}\sum {_{j}SD(i,j)}}}, &\text{\(SD(i,j)\neq{0}\)} \\ 0, & \text{otherwise} \end{array} \right.  $$

11 $$ LD(i,j)=\left\{ \begin{array}{cc} \frac{LD^{\prime }(i,j)}{\sum {_{i}LD^{\prime }(i,j)}}, &\text{\(SD(i,j)\neq{0}\)} \\ 0. & \text{otherwise} \end{array} \right.  $$

### Construction of the heterogeneous network

We first use the two matrices *LD*, *LL* to construct two networks, namely a disease similarity network, and an lncRNA similarity network. In the lncRNA similarity network, the edge between *l*(*i*) and *l*(*j*) is weighted by the similarity value of these two lncRNAs.

Likewise, in the disease similarity network, the edge between *d*(*i*) and *d*(*j*) is weighted by the similarity value of these two diseases.

Besides, the lncRNA-disease association network can be modeled as a bipartite graph. In this graph, the heterogeneous nodes correspond to either lncRNA or disease, and edges denote the presence or absence of the associations between them. If there is a known association between disease *d*(*i*) and lncRNA *l*(*j*), the weight of the edge is 1; otherwise it is 0. We divide the nodes of the heterogeneous network into two types. Those nodes connecting the lncRNA similarity network with the disease similarity network are called bridging nodes, and the other nodes are named internal nodes [21].

**Fig. 4:**
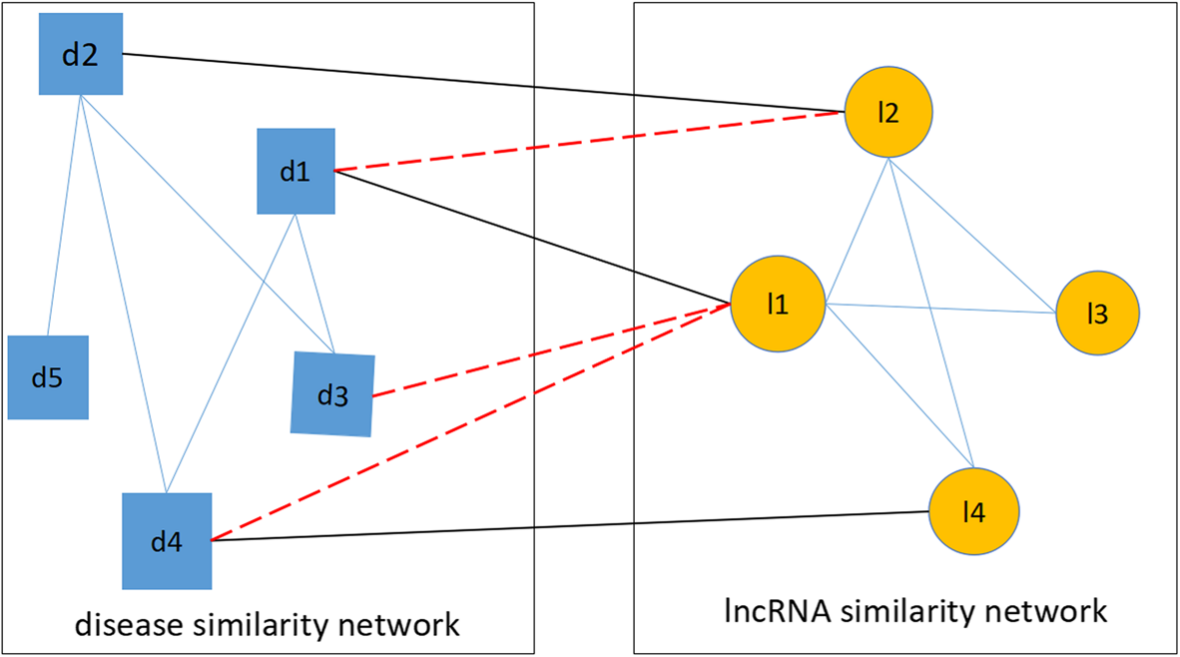
An illustrative example of heterogeneous network. An illustrative example of heterogeneous network. The squares indicate the nodes of diseases, and the edges between disease nodes describe the weights determined by the similarity value between diseases. The circles indicate the nodes of lncRNAs, and the edges between lncRNAs nodes describe the weights determined by the similarity value between lncRNAs. The edges between diseases and lncRNAs indicate the known lncRNA-disease associations, and the dashed lines indicate the predicted potential lncRNA-disease relationship

The heterogeneous network can be constructed by connecting the lncRNA similarity network and the disease similarity network via the known lncRNA-disease associations. A simple example of a heterogeneous network is illustrated in Fig. 4.

### Lap-BiRWHLDA

**Fig. 5:**
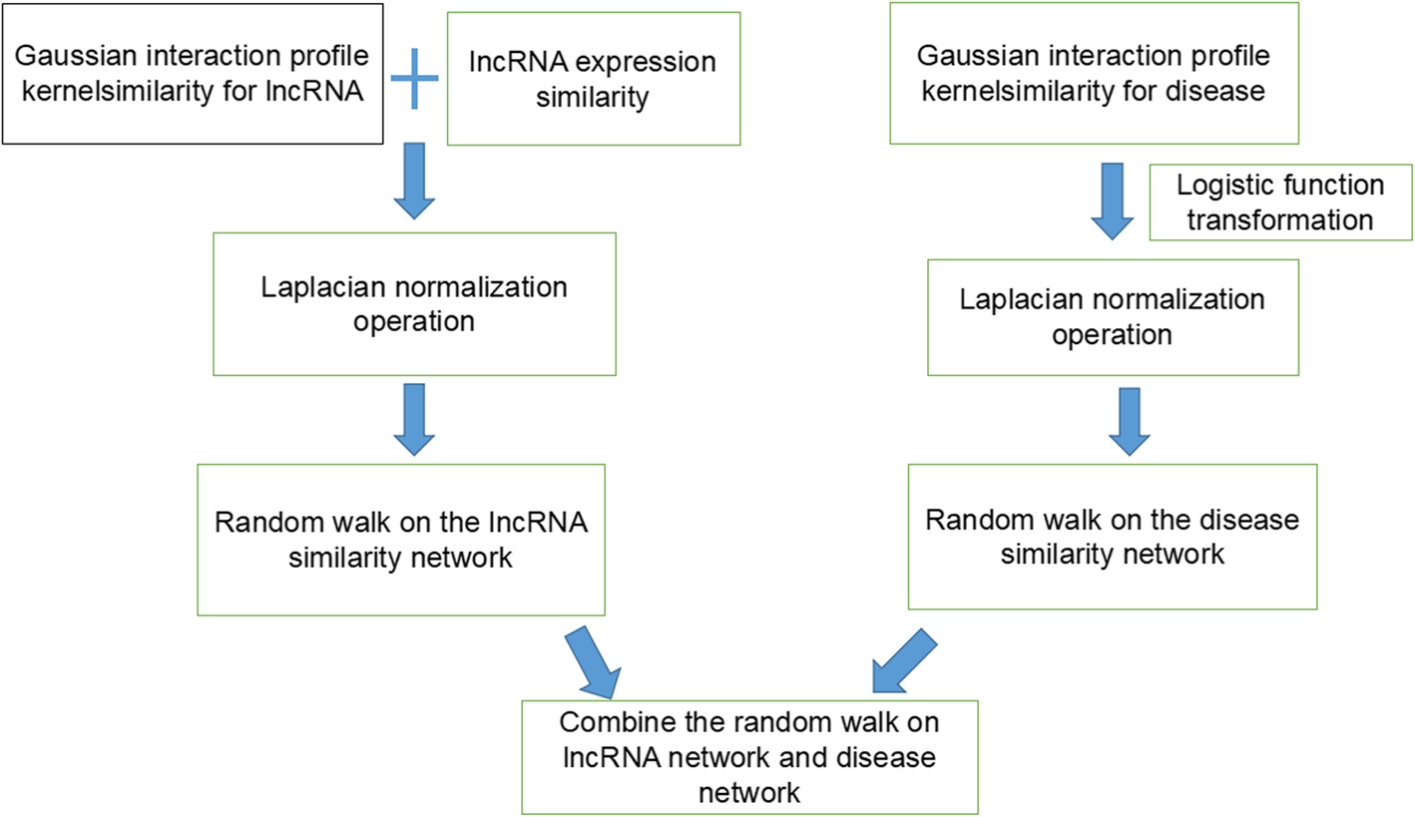
The flowchart of Lap-BiRWHLDA

In this study, we develop a novel computational method called Lap-BiRWHLDA to predict human lncRNA-disease associations. Figure 5 shows the flowchart of Lap-BiRWHLDA. Firstly, lncRNA similarity and disease similarity can be calculated based on the known lncRNA-disease associations taken from the LncRNADisease database. Secondly, the global heterogeneous network is built by combining the lncRNA similarity network, the disease similarity network and the lncRNA-disease association network. Finally, the bi-random walk algorithm is performed on the heterogeneous network to obtain the association probability scores between lncRNAs and diseases.

Suppose a random walker can jump from *d*(1) to *l*(1) and then to *l*(2). We can take *d*(1) as the starting node for the random walk. To simulate this process, we apply a random walk on the lncRNA similarity network. The iterative process can be described as

12 $$ {RT}_{L}^{t}=\alpha {{RT}_{L}^{t-1}}LL+(1-\alpha){Rt}_{0}.  $$

Similarly, we can also apply a random walk on the disease similarity network as follows:

13 $$ {RT}_{D}^{t}=\alpha {LD}{RT}_{D}^{t-1}+(1-\alpha){Rt}_{0},  $$

where *α* is a parameter to control the restart probability for the random walker, ${RT}_{L}^{t}$ is the predicted association between lncRNA *l* and disease *d* in the *t*-th iteration, ${RL}_{D}^{t}$ is the predicted relevance between disease *d* and lncRNA *l* in the *t*-th iteration, with

14 $$ {RT}_{L}^{0}={RT}_{D}^{0}={Rt}_{0}=A/sum(A).  $$

After the bi -random walks in the disease similarity network and in the lncRNA similarity network in the *t*-th step, Lap-BiRWHLDA further combines $ {RT}_{L}^{t}$ and ${RT}_{D}^{t}$ into *RT*^*t*^ as follows:

15 $$ {RT}_{L}^{t}={RT}_{D}^{t}=RT^{t}=\frac{{RT}_{L}^{t}+{RT}_{D}^{t}}{2}.  $$

After several steps, when the change between *RT*^*t*+1^ and *RT*^*t*^ is less than 10^−10^, we obtain the steady prediction score matrix *RT*, where *RT*(*i*,*j*) is the probability of potential association disease *d*(*i*) and lncRNA *l*(*j*).

## Abbreviations

AUC: Areas under ROC curve
Lap-BiRWRHLDA: Laplacian normalization and bi-random walks on heterogeneous networks for predicting lncRNA-disease associations
LOOCV: Leave-one-out cross-validation
ROC: Receiver-operating characteristics
